# beRBP: binding estimation for human RNA-binding proteins

**DOI:** 10.1093/nar/gky1294

**Published:** 2018-12-27

**Authors:** Hui Yu, Jing Wang, Quanhu Sheng, Qi Liu, Yu Shyr

**Affiliations:** 1 Center for Quantitative Sciences, Vanderbilt University Medical Center, Nashville, TN 37232, USA; 2 Department of Biostatistics, Vanderbilt University Medical Center, Nashville, TN 37203, USA

## Abstract

Identifying binding targets of RNA-binding proteins (RBPs) can greatly facilitate our understanding of their functional mechanisms. Most computational methods employ machine learning to train classifiers on either RBP-specific targets or pooled RBP–RNA interactions. The former strategy is more powerful, but it only applies to a few RBPs with a large number of known targets; conversely, the latter strategy sacrifices prediction accuracy for a wider application, since specific interaction features are inevitably obscured through pooling heterogeneous datasets. Here, we present beRBP, a dual approach to predict human RBP–RNA interaction given PWM of a RBP and one RNA sequence. Based on Random Forests, beRBP not only builds a specific model for each RBP with a decent number of known targets, but also develops a general model for RBPs with limited or null known targets. The specific and general models both compared well with existing methods on three benchmark datasets. Notably, the general model achieved a better performance than existing methods on most novel RBPs. Overall, as a composite solution overarching the RBP-specific and RBP-General strategies, beRBP is a promising tool for human RBP binding estimation with good prediction accuracy and a broad application scope.

## INTRODUCTION

RNA-binding proteins (RBPs) are a broad class of proteins, which coordinate co- and post-transcriptional gene regulation through binding to premature or mature mRNAs (1,2). Since the early discovery of heterogeneous nuclear ribonucleoproteins, various RNA-binding domains have been characterized, and many RBPs have been identified (3,4). As important co- and post-transcriptional regulators, RBPs are involved in many human diseases, such as neurologic disorders and cancers (5). Recent pan-cancer studies have even found that RBPs possess more striking expression aberration than transcription factors, suggesting that RBPs play an important role in cancer pathogenesis (6,7).

Identifying RBP targets and building RBP–RNA regulatory networks are critical for understanding the RBP function. However, predicting RBP–RNA interactions remains challenging due to interaction complexity and our limited knowledge of how RBPs recognize their targets. With a very limited number of known RNA targets, researchers pooled all known RBP–RNA interactions to train a universal classifier, in an attempt to learn the general interaction features applicable to all RBPs (8–11). For instance, Support Vector Machine or Random Forest was employed to develop a classifier involving >100 features derived from all known RBP targets. Recently, a statistical test-based method, RBPmap, was proposed for distinguishing potential target sequences of RBPs (12). Although RBPmap takes advantage of each RBP’s sequence binding preference, it still provides a generic strategy in which one common model is used to predict targets for all RBPs. Overall, such RBP generic strategy is pragmatic and successful, but has limited prediction accuracy, since specific binding properties are inevitably obscured through pooling heterogeneous datasets.

In the past few years, the rapid development of high-throughput techniques has greatly expanded our knowledge of RBPs. *In-vitro* (SELEX (13) and RNAcompete (14)) and *in-vivo* experiments (RIP-chip (15), RIP-seq (16) and CLIP-seq (17)) streamlined RBP-bound RNA extraction and detection at the transcriptome scale. These new techniques have been used to identify binding targets of individual RBPs in a high-throughput manner (18–20). As a result, a large number of targets have been discovered for a few RBPs, which are collected into databases such as RBPDB (21), doRiNA (22) and AURA (23). In addition to the expansion of RBP-specific known targets, our knowledge of RBP-binding motifs has greatly improved. Based on abundant target sequences for an individual RBP, a degenerate RNA segment (usually 4–7 nucleotides long) can be profiled as the RBP-specific binding preference. Recently, systematic RNAcompete experiments have determined RNA sequence preferences for 207 RBPs, including 85 human RBPs (24).

The accumulation of target RNAs and binding motifs for individual RBPs enable the development of RBP-specific target prediction methods. In designing the RBP target prediction method Oli (25), it has been pointed out that ‘it is reasonable to train one Support Vector Machine per RBP in order to model its specific binding properties.’ Oli resembles the earlier RBP-generic methods in many ways, but it develops one classifier for each RBP-specific training datasets. Lately, DeepBind employs deep learning to build a specific model for each RBP separately (26). iONMF integrates multiple data sources, such as gene region type, sequence motifs, gene annotation, RNA secondary structure and RBP co-binds, to discover RNA binding of each specific RBP (27). iDeep, built upon iONMF, proposes a deep-learning based framework to predict RBP–RNA interaction (28). Such RBP-specific strategy has the potential to capture unique RNA binding patterns inherent in each RBP, but prediction accuracy is highly dependent on the size of each training dataset. Since only a few RBPs have sufficient data to warrant the prediction power, the RBP-specific strategy is not applicable to the vast RBPs with very few or null known targets.

Here, we propose beRBP (‘Binding Estimation for human RBPs’) to predict human RBP targets, a dual approach overarching the RBP-specific and RBP-General strategies. ‘Specific models’ were built for 29 human RBPs, each of which had a sizeable number of known binding targets. Beyond that, a ‘General model’ was established for handling any RBPs with little or no target information but known binding preferences. The Specific and General models both compared well with existing methods on three benchmark datasets compiled from AURA (23), ENCODE eCLIP (29), and doRiNA (22). Notably, the beRBP-General model performed better than DeepBind and RBPmap on most novel RBPs, none of whose targets were used to build the model. Compared with DeepBind models for 80 human RBPs and RBPmap predictions for 94 human RBPs, beRBP provides general predictions for 143 human RBPs. With a general strategy, although both RBPmap and beRBP-General can be applied to any RBPs with known PWMs, RBPmap requires the motif length to be 4–10 bp long, while beRBP-General has no restriction on the motif length. In addition, beRBP webserver provides general predictions for user-provided PWM or even RBP sequence, from which PWMs are inferred based on the finding that two proteins sharing >50% sequence identity on RBDs (RNA Binding Domains) are likely to have similar motifs (24). Overall, beRBP is a powerful tool for predicting RNA targets of human RBPs with outstanding prediction accuracy and a broad application scope. beRBP is available at http://bioinfo.vanderbilt.edu/beRBP/.

## MATERIALS AND METHODS

### Sequence and structure features

**Figure 1.**
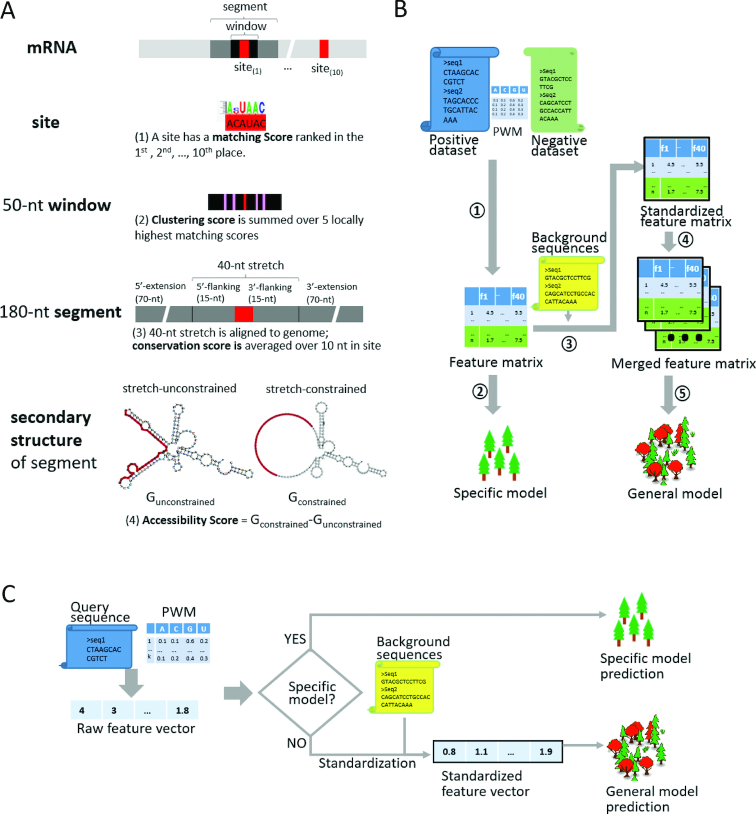
Schema of beRBP. (**A**) Four types of features. (1) Matching score is calculated as the high-scoring match to a given PWM; (2) clustering score is estimated by summing the rank-weighted five locally matching scores of a 50-nt window centered on the putative binding site; (3) conservation score is quantified by an average of the conservation scores of ten consecutive positions starting at the putative binding site; (4) spatial accessibility score is calculated by the difference in the minimum free energy between unconstrained and constrained secondary structures for the 180-nt segment, which is extracted by extending the binding site (∼10 nt) at both sides with 15-nt (immediate flanking) stretches and 70-nt (further flanking) sequences. (**B**) beRBP-Specific models and beRBP-General model. Given a PWM, positive and negative sequences are converted into a feature matrix (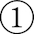), and a specific Random Forest model is trained over the feature matrix (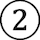). The raw feature matrix is standardized against background sequences (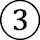), and feature matrices for RBPs are pooled together (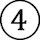) to build the General model (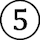). (**C**) beRBP webserver. Users can upload one or multiple query sequences, choose one, multiple or all RBP:PWM(s) for specific or general predictions. beRBP will determine whether RBP(s) binding to RNA sequence(s) of interest.

Given a candidate RNA sequence and an RBP motif represented by a position weight matrix (PWM), four types of features were generated to consider motif match, sequence environment, structural accessibility and evolutionary conservation of each putative binding site (Figure 1A).

### Matching score (MS)

The candidate sequence was scanned to identify the top *N* best matches to the given PWM. The matching score (${\rm{MS}}$) of the subsequence starting from position *i* was calculated according to Equation (1), where *k* is the motif length, *N_j_* the *j*th (*i* ≤ *j* ≤ *i* + *k* – 1) nucleotide in the candidate sequence $( {{N_j} \in ( {{\rm{A}},{\rm{C}},{\rm{G}},{\rm{T}}/{\rm{U}}} )} )$, and ${{\rm{f}}_{{\rm{N}}_{\rm{j}}^{{\rm{j}} - {\rm{i}} + 1}}}$ the frequency of the *j*th nucleotide at the *j* − *i* + 1 position in the given PWM. To find the optimal *N*, beRBP performance using different number of best matching sites (*N* = 3, 5, 10, 15 or 20) were evaluated based on the binding target sets of PUM2, QKI, and ELAVL1 from a related study (25). Across all three datasets, the prediction performance improved consistently as the number of matching sites increased from 3 to 10, while it became stable or even decreased when the parameter further increased to 15 or 20 (Supplementary Figure S1). This suggested that the top 10 best matches contribute most to the binding prediction. Therefore, the top 10 matching sites were considered as putative binding sites, and the corresponding matching scores were denoted as MS1, MS2, …, and MS10.

(1) \begin{equation*}{\rm MS} = \sum\nolimits_{{{j}} = {{i}}}^{{{i}} + ({{k}} - 1)} {\left( {{{{f}}_{{{N}}_{{j}}^{{{j}} - {{i}} + 1}}}} \right)} \end{equation*}

### Clustering score (CS)

Besides PWM match, the sequence environment of each putative binding site was also considered. The clustering propensity around each site was estimated by calculating the matching score of a 50-nt window centered on the site as previously described by RBPmap (12). In detail, five locally maximum matching scores within the 50-nt window were first identified and ranked. Each matching score was then weighted by its rank and the clustering score $CS{\rm{\ }}$was calculated by summing the rank-weighted matching scores as Equation (2), where${\rm{\ }}{{\rm{S}}^r}$ denotes the matching score (defined in Equation 1) ranked at the top *r*th place and ${2^{ - r}}$ denotes the rank-based weight.

(2) \begin{equation*}{\rm{CS}} = \sum\nolimits_{{\rm{r}} = 1}^5 {{2^{ - {\rm{r}}}}{S^r}} \end{equation*}

### Spatial accessibility (Gacc)

To assess the spatial accessibility of each putative binding site, a 180-nt segment was extracted by extending the binding site (∼10 nt) at both sides with 15-nt (immediate flanking) stretches and 70-nt (further flanking) sequences (180 = 10+2 × 15+2 × 70). Previous studies have discovered that not only the target site but also 3∼15 flanking nucleotides should be considered to give a more accurate accessibility quantification (30,31). To be safe, we chose to extend the site by 15 flanking nucleotides. Since there is a low probability of secondary structure base-pairing interactions between nucleotides that are separated by more than 70 nucleotides (31), we further extended 70 bases at both sides to predict the RNA fold structure. Using a similar strategy as previously described (31), an accessibility score $Gacc$for each putative binding site was calculated as the difference in free energy of ensemble structure between the original segment (*G*_unconstrained_) and a constrained segment (*G*_constrained_) (Equation 3). The constrained segment, which had the same sequence as the original segment, was subject to a folding constraint that the 40-nt core stretch must remain unpaired. Program ‘RNAfold’ from the toolkit ViennaRNA (v2.1.9) (32) was employed for RNA folding and free energy estimation.

(3) \begin{equation*}{\rm Gacc} = {{{G}}_{{\rm{constrained}}}} - {{{G}}_{{\rm{unconstrained}}}}\end{equation*}

### Conservation score (Csrv)

Additionally, the evolutional conservation of each putative binding site was considered. The 40-nt core stretch was aligned against the human reference genome using MegaBlast (33). Based on the UCSC track ‘phyloP100way’, the conservation score (Csrv) for the binding site starting from position *i* was quantified by an average of the conservation scores of ten consecutive positions (*c_j_, i* ≤ *j* ≤ i + 9) (Equation 4).

(4) \begin{equation*}{\rm Csrv} = \sum\nolimits_{{{j}} = {{i}}}^{{{i}} + 9} {{{{c}}_{{j}}}} \end{equation*}

In summary, there are four types of features for each putative binding site, namely matching score (MS), clustering score (CS), spatial accessibility (Gacc), and conservation (Csrv). With ten putative binding sites, ten matching scores (denoted as MS1, MS2, …, MS10), ten clustering scores (CS1, CS2, …, CS10), ten accessibility scores (Gacc1, Gacc2, …, Gacc10), and ten conservation scores (Csrv1, Csrv2, …, Csrv10) would be obtained. That is, each candidate sequence was encoded into a vector of totally 40 features, which serves as the feature matrix of the Random Forest model.

### Post-scoring standardization

Each RBP has its unique binding preference, represented as a PWM. Highly dependent on PWMs, features are not directly comparable across RBPs. A post-scoring standardization step, which removes the dependence of features on PWMs, is expected to break through barriers caused by RBP binding specificity. Here, z-transformation was used to standardize feature scores specific to a given PWM. To do this, 21 147 randomly chosen 3′-UTR sequences were used as the background set. For each background sequence, a vector consisting of all aforementioned 40-feature scores on the given PWM was calculated. For the feature *j* (1 ≤ *j* ≤ 40), scores of background sequences formed an empirical distribution, from which its mean *M_j_* and standard deviation *S_j_* were derived. Based on the background distribution, a raw feature score *f_ij_* (for candidate sequence *i* and feature *j*) was transformed into a standardized z-score, *z_ij_* (Equation 5).

(5) \begin{equation*}{{{z}}_{{{ij}}}} = \frac{{\left( {{{{f}}_{{{ij}}}} - {{{M}}_{{j}}}} \right)}}{{{{{S}}_{{j}}}}}\end{equation*}

### RBP motifs and benchmark datasets

The binding preferences of RBPs, represented as PWMs, were retrieved from cisBP–RNA database (24) (build 0.6; http://cisbp-rna.ccbr.utoronto.ca/), which collected RNAcompete-recognized RBP-binding motifs (24), as well as other motifs inherited from an earlier database RBPDB (21). Since RNAcompete motifs dominate in cisBP–RNA, we preferred RNAcompete motifs over others for each RBP. Non-RNAcompete motifs were chosen only when RNAcompete motifs were unavailable. In this way, some RBPs have one motif, while other RBPs possess multiple motifs.

Experimentally validated target sequences (3′-UTRs) for human RBPs (positive datasets) were downloaded from AURA (v2, 8/5/2015; http://aura.science.unitn.it/), which is a manually curated and comprehensive catalog of human UTRs bound by regulators, including RBPs. Target sequences shorter than 150 nucleotides were removed. After the filtration, CIRBP and NCL have very few target sequences, 64 and 97, respectively. For those RBPs with >6000 target sequences, like ELAVL1 and IGF2BP1, module ‘cd-hit-est’ from the web-service CD-HIT (34) were implemented to cluster sequences of 90% or higher similarity, by which redundancy was removed and the size of the dataset was reduced. The size of positive dataset for each RBP was shown in Supplementary Table S1.

Designation of negative datasets is generally problematic since we don’t have experimental negatives. A previous study has demonstrated that random sequences can provide a good approximation when no experimental negatives are available, which showed highly correlated performance between experimental negatives and random negatives (*R* = 0.99) (25). Following the idea, we randomly chose 3000 sequences from the 3′-UTR pool as pseudo negatives. After removing short sequences (<150 nt) and those overlapping with positive sequences, the size of the negative dataset for each RBP varied slightly (Supplementary Table S1). Repeatedly, we generated five random negative datasets, where each negative were paired with the positive to establish the dataset for each RBP.

### beRBP-specific and beRBP-General models

A specific model was built for one RBP if its binding preference was available and it had sufficient number of known targets (>100 before length filtration) in AURA. Among the RBPs covered by AURA, we obtained motifs of 28 human RBPs (Supplementary Figure S2A) from cisBP–RNA and the motif of PUM2 from a published study (31). Therefore,we developed beRBP-Specific models for 29 RBPs in total. Technically, we built a specific model for each unique RBP:PWM combination. Since some RBPs had multiple PWMs, we actually built 37 Specific models for 29 RBPs (Supplementary Table S1). beRBP performance was evaluated on five datasets (the positive paired with each negative from five random negatives, Materials and Methods) using an out-of-bag strategy (35). The training and predicting processes were implemented using R package ‘randomForest’ (36) (Figure 1B).

Using the post-scoring standardization described above, we pooled known targets from different RBPs to build an RBP-generic model. The raw feature scores of each RBP were standardized by z-transformation against a background dataset; afterwards, standardized data matrices from different RBPs were used to train the model which involved 141 143 positive sequences. 282 286 sequences, randomly sampled from the 3′UTR pool, were used as the negative dataset, leading to a 1:2 positive–negative ratio. Currently there is no consensus on how to select the optimal positive-negative ratio. Although it is recommended to use equal portion of positive and negative samples in machine-learning approaches, this practice generally does not give good results in the real life because it doesn’t reflect the ratio in reality. In the eCLIP dataset, which kind of reflect the ratio in reality, most RBPs only have 6000–10 000 binding sites out of more than 20,000 genes (29). Considering there are generally more non-binding events than RBP binding event, we thought that a 1:2 positive-negative ratio instead of 1:1 would achieve good performance in the whole-transcriptome scan. A Random Forest classifier, termed the ‘General model’ henceforth, was trained to capture the general feature of RBP–RNA interactions beyond PWM confinement (Figure 1B). Unlike specific models, beRBP-General model can be applied to any RBPs as long as their binding preferences are available. cisBP–RNA included motifs for 153 human RBPs; after removing deprecated IDs, 143 human RBPs were left. beRBP provide general predictions for all 143 human RBPs.

beRBP models are available at http://bioinfo.vanderbilt.edu/beRBP/. beRBP enables binding discovery on one/multiple RNA sequences for 29 RBPs/37 PWMs (Specific models), 143 RBPs/175 PWMs (the General model), and any RBPs with user-provided PWMs or RBP sequences (the general model). beRBP allows users to select one RBP/PWM, multiple RBPs/PWMs, or all RBPs/PWMs, which is very useful for screening RBP(s) binding to RNA sequence(s) of interest (Figure 1C).

### Whole-transcriptome target scan

In order to evaluate beRBP performance at the whole-transcriptome scale, beRBP was applied to scan all human 3′-UTR sequences for binding prediction. Targets from ENCODE eCLIP data (https://www.encodeproject.org) were used as the gold standard, which utilizes enhanced CLIP technologies to identify reliable in vivo RBP binding targets (Supplementary Table S2) (29). Only peaks falling into, or overlapping with 3′-UTR regions were considered. Common peaks (i.e. peaks located within the same 3′-UTR) identified from two replicate experiments were treated as true positives. Since some eCLIP targets were also included in AURA and thus already used to build the model, those common targets were excluded to make a fair and unbiased comparison with existing methods, which removed 0.4–24.8% of eCLIP targets. Although eCLIP provides binding targets for 115 human RBPs, only 25 RBPs have PWMs available in cisBP–RNA (Supplementary Figure S2B). In addition, we added the PWM of another RBP (PUM2) from a published study (31). Therefore, beRBP-General was implemented for these 26 RBPs since PWM is a required input. Among 26 RBPs, 17 RBPs have prebuilt beRBP-specific models, 25 met the motif length requirement of RBPmap (4–10 nt), and 19 have DeepBind models available (Supplementary Figure S2B).

### Binding prediction on any RNA regions

To further evaluate beRBP performance on any RNA segments without limiting to 3′ UTR regions, binding targets from doRiNA were used to establish the gold standard, which collects in vivo binding sites of individual RBPs from CLIP-seq studies (22). We included all binding sequences in any RNA regions, including 5′-UTRs, introns, exons, and 3′-UTRs. As described in a previous study (24), sequences with doRiNA score in the top five percentile were treated as binding targets/positive sequences. When necessary, the percentile cutoff was relaxed to include a maximum of 1000 sequences. Sequences shorter than 300 nt were extended symmetrically in both directions to 301 nt. Excessively long sequences (>21 000 nt) were removed. In both the upstream and downstream of 300-bp from each positive sequence, 301-nt sequences were extracted as negative data. The non-redundant positive and negative sequences were compiled into the benchmark. The performance of beRBP, RBPmap and DeepBind were evaluated on 14 human RBPs, which had binding data in doRiNA, PWMs available in cisBP–RNA and were also covered by DeepBind (Supplementary Figure S2C).

## RESULTS

### beRBP-Specific models achieved good performance for RBP binding prediction

beRBP-Specific models for 29 human RBPs/37PWMs were trained using Random Forests based on the positive dataset from AURA and five negative datasets from random sequences (Materials and Methods). The performance was estimated by the AUC (area under receiver-operating-characteristic curve) calculated from out-of-bag votes, which is the prediction on each sample using only the trees that do not have the sample in their bootstrap procedures. beRBP was compared with three latest methods, RBPmap (12), DeepBind (26) and iONMF (27). RBPmap provides a universal classifier for all RBPs, while DeepBind and iONMF belong to the RBP-specific category. Unlike beRBP, RBPmap, and DeepBind providing pre-built models, iONMF equips users an algorithm to construct prediction models. Additionally, while other methods take sequences as the input, iONMF requires users to generate multiple biological feature matrices by themselves, such as region type, structure, and GO annotations. iONMF was implemented for all 29 RBPs/37 PWMs, while RBPmap was applied to 27 RBPs/34 PWMs except IGF2BP1, NCL and ZFP36_1, since their motifs length exceed the requirement of 4–10 nt. Although DeepBind provided models for 80 human RBPs out of totally 194 RBP models, only 19 RBPs have binding data in AURA (Supplementary Figure S2A). Therefore, the performance of DeepBind on these 19 RBPs was estimated and was compared to beRBP.

**Figure 2.**
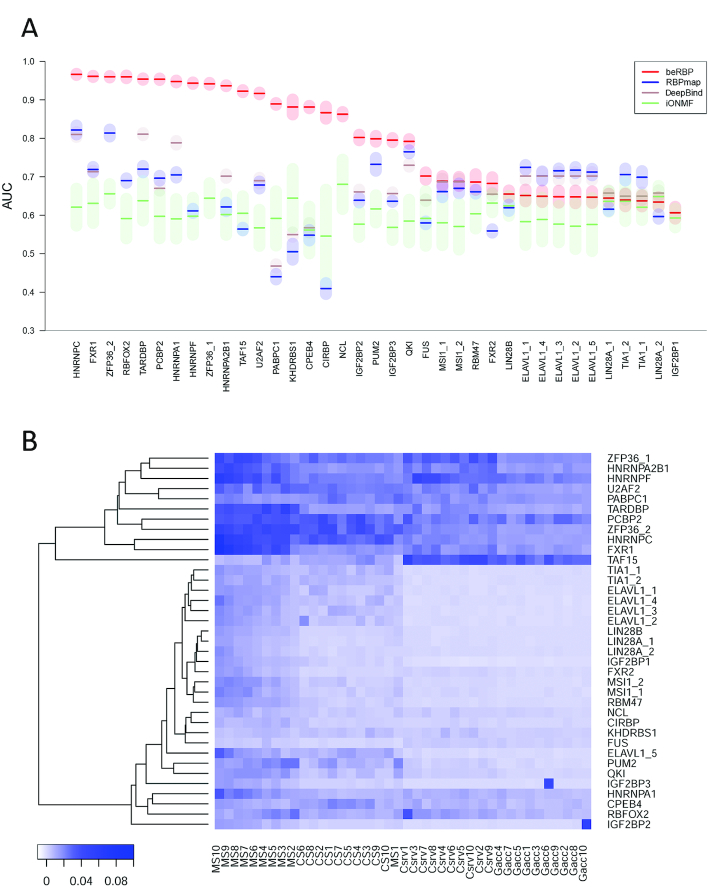
(**A**) Comparison between beRBP-specific models, RBPmap, DeepBind and iONMF. Y-axis lists AUC values on one positive and five negative datasets for the 37 RBP:PWM combinations. (**B**) The heatmap of the importance of each feature (X-axis) in each RBP-specific model (Y-axis). X-axis list the 40 features, ordered decreasingly from left to right by their average importance across the 37 models.

Overall, beRBP-Specific models achieved better performance for most RBPs/PWMs, with AUC values ranging from 0.61 to 0.97 and a median value of 0.80 (Figure 2A and Supplementary Table S1). In contrast, RBPmap had AUC values between 0.41 and 0.82 with a median value of 0.67; DeepBind obtained AUC values of 0.47 to 0.81 with a median value of 0.67; and iONMF got AUCs ranging from 0.55 to 0.68 with a median value of 0.6. Specifically, beRBP outperformed RBPmap for 27 of the 34 RBP:PWM models, especially for CIRBP, CPEB4, KHDRBS1 and PABPC1, while RBPmap obtained slightly higher prediction accuracy than beRBP for ELAVL1 and TIA1. beRBP achieved higher or roughly similar performance than/to DeepBind for all RBPs except ELAVL1. beRBP was superior to iONMF for all RBPs except LIN28A and TIA1, for which two methods obtained comparable performance.

In our study, due to limited targets in AURA, negative instances heavily outnumber the positive ones for some RBPs, such as CIRBP and KHDRBS1. It has been found that AUC does not correlate well with the positive/negative predictive values in those severely imbalanced datasets. Thus a more robust measure, Area Under Precision-Recall curve (AUPRC) was recommended (25,37). On this secondary evaluation metric of AUPRC, beRBP outperformed RBPmap, DeepBind, and iONMF as well (Supplementary Figure S3). For beRBP, AUPRC values were significantly correlated with AUC values across the 37 beRBP-Specific models (Spearman correlation coefficient *r* = 0.695, *P* = 3.8E–6; Supplementary Figure S4) (Supplementary Table S1). As highlighted in Supplementary Table S1, 13 of the 37 beRBP-Specific models, including FXR1, HNRNPA1, HNRNPA2B1, HNRNPC, HNRNPF, PABPC1, PCBP2, RBFOX2, TAF15, TARDBP, U2AF2, ZFP36_1 and ZFP36_2, had the highest prediction accuracy in terms of both AUC and AUPRC (AUC > 0.85 and AUPRC > 0.85).

The importance of each feature was assessed by the decrease of predictive power in the absence of the feature. Averaging the ranks of importance across all 37 Specific models, we found that the contribution of each feature to the prediction power decreased primarily by the type of features in the following order: matching (‘MS’), clustering propensity (‘CS’), conservation (‘Csrv’), and accessibility (‘Gacc’, Figure 2B). It is expected and reasonable that matching scores precede other types of features, since clustering, conservation, and accessibility scores are all dependent on the potential binding sites. Surprisingly, the highest matching score (MS1) was not ranked as the most important feature. The exceptionally low importance of MS1 implied that a naïve prediction based on merely the best matching score might not be effective. Neighboring context combined with the derived secondary features also contribute to RBP binding. Based on the feature importance profiles, the 37 Specific models were clustered into two groups. In the clustering tree, the upper group, consisting of 11 RBPs, possesses a higher feature importance than the bottom group (Figure 2B). All 11 models are among the 13 RBPs with both the highest AUC and AUPRC values.

### The General model showed comparable accuracy to Specific models

**Figure 3.**
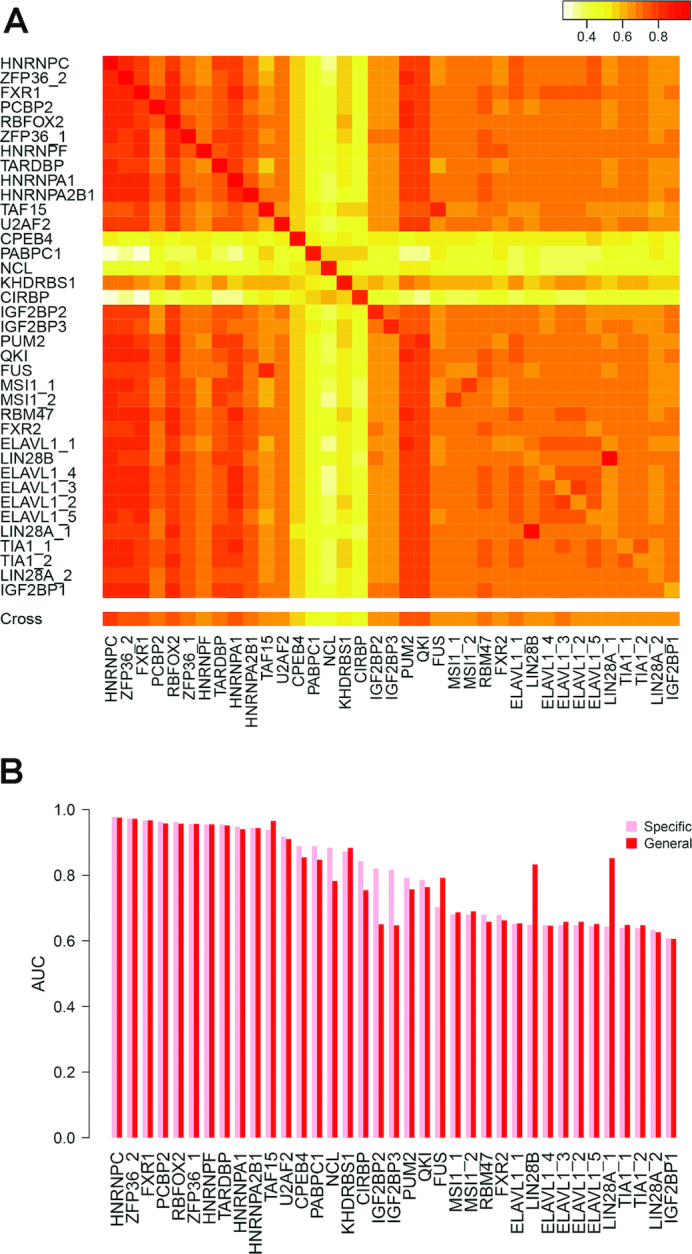
(**A**) The heatmap of AUC values from cross-RBP prediction, where the model trained by one RBP (on the row) is used to predict targets of another RBP (on the column). The average AUC of cross-RBP predictions on each RBP was summarized in the ‘Cross’ row at the bottom. (**B**) Comparison between beRBP-Specific models (pink) and the beRBP-General model (red).

We assumed that the post-scoring standardization would make features comparable across RBPs, and thus a model trained by standardized features pooled from all RBP–RNA interactions, was expected to capture common patterns of RBP recognizing targets. To test this assumption, cross-prediction performance was evaluated; that is, the model trained by one RBP was used to predict targets for another RBP. Overall, the cross-RBP models showed good performance for most RBPs except CPEB4, PABPC1, NCL, KHDRBS1 and CIRBP (Figure 3A). The exceptionally low cross-prediction performance of those RBPs was most likely due to the small size of their positive datasets. We found that the cross-prediction performance was independent of motif similarity (Supplementary Figure S5, Pearson correlation *r* = 0.25, *P* = 0.167). For example, HNRNPC and FXR1 have very different motifs, however, the model trained by HNRNPC was successfully applied to predict targets of FXR1, and vice versa (AUC = 0.8) (Figure 3A). As another example, PCBP2 binding motif is dissimilar to all other RBPs, but the model trained by PCBP2 achieved high cross-prediction performance for other RBPs (Figures S5 and 3A). The good performance of cross-RBP models confirmed that there was some kind of commonalities shared by RBPs in recognizing targets. The disassociation of cross-prediction performance with the motif similarity further suggested that the common features were beyond the simple motif match.

Specific models achieved a high prediction accuracy than the General model on NCL, CIRBP, IGBF2BP2 and IGF2BF3. The high performance of the General model for LIN28B and LIN28A_1 (Figure 3B) was most likely to be overestimated. Since LIN28B and LIN28A_1 had the same motif and shared most targets (>90%), there was overlap between training and testing datasets when the General model trained by targets of all RBPs except LIN28B/LIN28A_1 was used to predict targets for LIN28B/LIN28A_1. Approximately, the General model trained by pooled RBP–RNA interactions achieved comparable performance to Specific models (Figure 3B). These results indicated the General model will be useful for predicting targets of novel RBPs with few or null available targets, thereby greatly expanding the utility of beRBP.

### beRBP performed best in whole-transcriptome target scan

**Figure 4.**
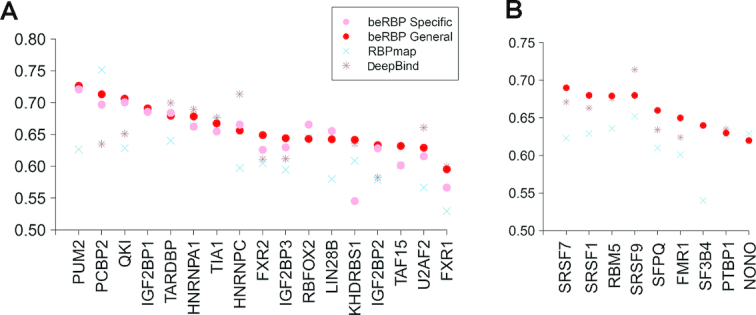
Comparison between beRBP, RBPmap and DeepBind on whole-transcriptome target scans for 26 RBPs. (**A**) Performance on 17 RBPs with beRBP-Specific models; (**B**) Performance on nine new RBPs, which were not included in building beRBP models.

To further evaluate the performance of beRBP-Specific and beRBP-General models at whole-transcriptome target scanning, the ENCODE eCLIP binding data were compiled as the gold standard (29). eCLIP used an enhanced CLIP-seq technique (eCLIP) to reliably identify in vivo binding at the transcriptome scale. Across the 17 RBPs with pre-built beRBP-Specific models, beRBP obtained a higher/similar prediction accuracy than/with RBPmap except PCBP2 (Figure 4A). beRBP achieved a better/comparable performance than/to DeepBind except TARDBP, HNRNPA1, HNRNPC and U2AF2 (Figure 4A and Supplementary Table S3). In terms of AUPRC, both beRBP-Specific models and beRBP-General model significantly outperformed RBPmap and DeepBind (*P* ≤ 0.01, one-sided Wilcoxon signed rank test; Supplementary Tables S3 and S5).

Notably, nine RBPs, namely FMR1, NONO, PTBP1, RBM5, SF3B4, SFPQ, SRSF1, SRSF7 and SRSF9, have not been included in building beRBP-Specific and beRBP-General models, which could be regarded as new RBPs to beRBP. The performance on these RBPs could suggest the applicability of beRBPs to any novel RBPs. Remarkably, beRBP-General model achieved a better/similar performance than/with DeepBind and RBPmap on all nine RBPs except SRSF9 (Figure 4B). RBPmap is essentially a RBP-generic method, while DeepBind uses a RBP-specific strategy, which employs known targets of one specific RBP to build a model for the RBP. Generally, RBP-specific method is more likely to achieve a better performance than RBP-generic approach since pooling RBPs would obscure RBP–RNA interaction features. As expected, DeepBind showed a higher accuracy than RBPmap. Surprisingly, beRBP-General model outperformed DeepBind on most new RBPs, further demonstrating the power of beRBP-General model on novel RBPs (Supplementary Table S4).

### beRBP performed best on RNA segments not limiting to 3′UTR

**Figure 5.**
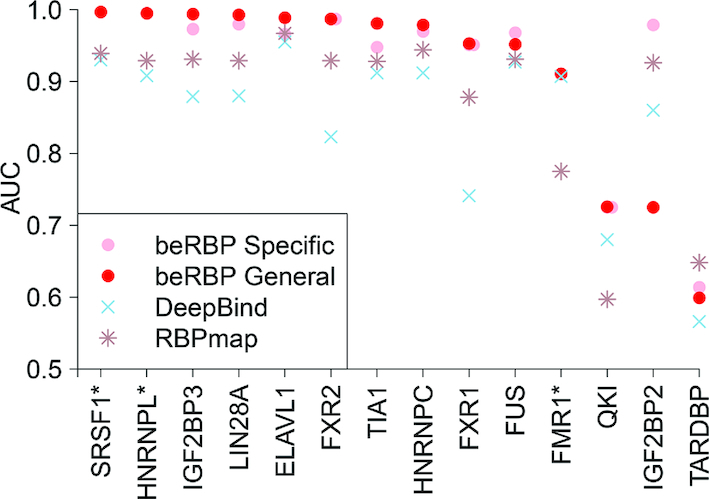
Comparison between beRBP, DeepBind and RBPmap for 14 RBPs on RNA segments without limiting to 3′UTR. *: new RBPs that were not included in building beRBP models.

RBPs not only bind to 3′-UTR, but also target other regions of RNA sequences, including 5′ UTR, intronic and exonic regions. To find out whether the model trained by 3′UTR regions can be applied to predicting binding in other regions, we further compared the performance of beRBP with DeepBind and RBPmap based on all target sites of 14 RBPs collected from doRiNA (Supplementary Table S6, Materials and Methods). Remarkably, beRBP outperformed DeepBind and RBPmap on all 14 RBPs except TARDBP (Figure 5). Even notably, three novel RBPs, FMR1, HNRNPL and SRSF1, which were not included in beRBP training, beRBP-General model achieved the highest prediction accuracy (labeled by * in Figure 5). These results based on unconstrained RNA segments demonstrated that the utility of beRBP models can be extended from 3′-UTR to any RNA regions.

## DISCUSSION

The existing methods for predicting RBP binding targets can be divided into two distinct categories, RBP-generic and RBP-specific. The generic strategy pooled RBP–RNA interactions to train a model applicable to any RBPs, while the specific strategy uses targets of each individual RBP to build a model tackling this RBP only. Generally, the specific strategy achieves better performance for those RBPs with sufficient number of known binding targets, while the generic strategy has a wider application scope. It is challenging to balance the tradeoff between prediction accuracy and application scope. Compared to existing methods, beRBP provided a composite solution overarching the RBP-specific and RBP-generic strategies. beRBP presented competitive specific models for some RBPs and a generic model for any RBPs, which achieved comparable performance with specific models.

beRBP enables high-quality target discovery for a broad spectrum of human RBPs. Although there are 1542 RBPs in the human genome (2), only ∼10% of RBPs have binding preference available in cisBP–RNA database. beRBP webserver provides general prediction for all these RBPs and also allows users to upload any PWMs of interest for General-model prediction. As more binding preferences of RBPs become available, beRBP can be easily expanded to those RBPs since PWM is the only requisite for beRBP-General prediction. Meanwhile, with the advance of techniques, more RBPs will accumulate sufficient training data to warrant the power of their specific models.

beRBP obtained higher/similar prediction accuracy than/with DeepBind, a deep-learning based method belonging to the specific category. The success of beRBP is partly attributed to combining multiple types of features rather than using sequence features alone like DeepBind. Clustering score, conservation scores and spatial accessibility scores helped improve the performance for the 13 best-performing Specific models of beRBP (Figure 2B). beRBP outperformed RBPmap as well, which used three common feature types except the structural accessibility. The improvement of beRBP over RBPmap mainly owes to the Random Forests that beRBP employs, which has demonstrated extraordinary performance in related works (8,9). Whereas RBPmap follows a simple non-parametric statistic test, beRBP is able to capture the complicated patterns among multiple features for RBP binding with the help of Random Forests.

In agreement with contemporary studies (12,25,26), our results indicate that the binding target predictability varies greatly across RBPs (Figures 2A and 4A). Some variability may be due to the difference in the quality of training datasets, which were derived from various CLIP-seq experiments. Relatedly, the size of RBP-specific training datasets and the positive-negative ratio would greatly affect prediction performance. Although a statistically significant negative correlation between the number of known binding sequences and AUC values was observed in our experiments with AURA datasets (Supplementary Figure S6), the dominant true negatives led to a high AUC value when the positive dataset was small. Caution should be paid for those beRBP-Specific models with limited or highly imbalanced training sequences, such as CIRBP, CPEB4, KHDRBS1 and NCL. For example, beRBP-Specific obtained low sensitivity for the whole transcriptome target screening for KHDRBS1 (Supplementary Table S2). Finding the optimal positive-negative ratio might improve their performance. On the other hand, the performance of beRBP-General would not be affected by the limited number of RBP-specific training sequences, and thus beRBP-General is recommended if a specific model was trained by limited targets.

It should be noted that each method performs well for a unique set of RBPs. For example, RNAcompete inventers predicted RBP targets using a principle of ‘strong motif match’, and the method was effective for QKI (AUC 0.93) but not for FUS (AUC 0.28) (24). With beRBP, both RBPs obtained decent AUC values (0.79 and 0.70). As another example, RBPmap predicted binding targets for QKI more accurately than for HNRNPA1 (12), whereas beRBP is more powerful for HNRNPA1 than for QKI. Considering all the factors that lead to variant predictability among RBPs, we have identified 13 beRBP-Specific models with both high and robust prediction accuracy (FXR1, HNRNPA1, HNRNPA2B1, HNRNPC, HNRNPF, PABPC1, PCBP2, RBFOX2, TAF15, TARDBP, U2AF2, ZFP36_1 and ZFP36_2). The 13 RBP models were trained by decent number of known targets (611–3889), and 11 of them share similar feature importance profiles.

## CONCLUSION

In this work, we proposed beRBP, an RBP target prediction algorithm that leverages the Random Forest classifier to analyze RNA sequence/structure features (motif matching, clustering, accessibility, and conservation). We built 37 Specific models, which demonstrated an overall superiority over existing methods on three benchmark datasets.

Beyond Specific models, beRBP pooled RBP–RNA interactions and trained a generic model (General model) to make binding estimation for any RBPs with known binding preferences. The prediction accuracy of beRBP-General model was comparable to that of Specific models. For most novel RBPs, beRBP-General model performed better than or comparable to existing methods. These results demonstrated that the beRBP-General is greatly useful for handling those RBPs for which it is unable to build powerful RBP-specific models due to limited known targets.

In summary, beRBP is a competitive tool for RBP binding estimation with outstanding prediction accuracy and broad application scope, holding promise for greatly expanding our knowledge about RBP–RNA interactions.

## DATA AVAILABILITY

beRBP is freely accessible at http://bioinfo.vanderbilt.edu/beRBP/. Source code, the whole transcriptome scan results, and all the training and testing datasets can also be downloaded from the website.

## Supplementary Material

Supplementary Data
