# Supplementary material for: beRBP: binding estimation for human RNA-binding proteins
Source: Nucleic Acids Res. 2018 Dec 27;47(5):e26. doi: 10.1093/nar/gky1294 (PMC6411931; doi:10.1093/nar/gky1294)
Supplement: Supplementary Data [file gky1294_supplemental_files.pdf]

beRBP: Binding Estimation for human RNA-Binding Proteins

Hui Yu[1§], Jing Wang[1,2§], Quanhu Sheng[1], Qi Liu[1,2*], and Yu Shyr[1,2*]

[1]Center for Quantitative Sciences, Vanderbilt University Medical Center, Nashville, TN 37232, USA.

[2]Department of Biostatistics, Vanderbilt University Medical Center, Nashville, TN 37232, USA.

Table S1. 37 beRBP-specific models.

**RBP model**[æ]	**Motif ID**[§]	**# targets**	**# pos_seqs**	**# neg_seqs**	**AUC**	**AUPRC**
CIRBP	M048_0.6	64	61	2565	0.87	0.66
CPEB4	M149_0.6	247	237	2558	0.88	0.80
ELAVL1_1	M031_0.6	7775*	7409	2202	0.65	0.81
ELAVL1_2	M108_0.6	7775*	7409	2202	0.65	0.81
ELAVL1_3	M112_0.6	7775*	7409	2202	0.65	0.81
ELAVL1_4	M127_0.6	7775*	7409	2202	0.65	0.81
ELAVL1_5	M232_0.6	7775*	7409	2202	0.65	0.81
FUS	M316_0.6	2615*	2494	2447	0.70	0.64
**FXR1**	M152_0.6	2900	2770	2438	0.96	0.97
FXR2	M020_0.6	3250*	3096	2414	0.68	0.67
**HNRNPA1**	M022_0.6	463	449	2554	0.95	0.89
**HNRNPA2B1**	M024_0.6	1775	1690	2483	0.94	0.94
**HNRNPC**	M025_0.6	4037	3856	2387	0.97	0.98
**HNRNPF**	M151_0.6	3655	3504	2417	0.94	0.97
IGF2BP1	M296_0.6	6538*	6258	2288	0.61	0.76
IGF2BP2	M032_0.6	6776*	6477	2269	0.80	0.93
IGF2BP3	M163_0.6	7953*	7611	2226	0.80	0.93
KHDRBS1	M160_0.6	117	111	2562	0.88	0.79
LIN28A_1	M035_0.6	4104*	3925	2389	0.65	0.68
LIN28A_2	M153_0.6	4104*	3925	2389	0.63	0.68
LIN28B	M035_0.6	4743*	4536	2361	0.66	0.73
MSI1_1	M040_0.6	3462*	3302	2408	0.69	0.69
MSI1_2	M167_0.6	3462*	3302	2408	0.69	0.69
NCL	M245_0.6	97	96	2567	0.86	0.70
**PABPC1**	M146_0.6	1035	997	2530	0.89	0.87
**PCBP2**	M043_0.6	3130	2995	2433	0.95	0.97
PUM2	Incarnato et al., 2013	3286	3143	2425	0.80	0.77
QKI	M046_0.6	841	803	2530	0.79	0.51
**RBFOX2**	M159_0.6	643	611	2536	0.96	0.92
RBM47	M234_0.6	6116*	5842	2294	0.69	0.79
**TAF15**	M316_0.6	3765	3611	2414	0.92	0.95
**TARDBP**	M074_0.6	1144	1090	2514	0.95	0.94
TIA1_1	M075_0.6	6386*	6077	2259	0.64	0.77
TIA1_2	M156_0.6	6386*	6077	2259	0.64	0.77
**U2AF2**	M077_0.6	1068	1013	2513	0.92	0.90
**ZFP36_1**	M269_0.6	4053	3889	2404	0.94	0.97
**ZFP36_2**	M350_0.6	4053	3889	2404	0.96	0.98

Bold and highlighted ones are 13 RBP models of the highest performance (AUC ≥ 0.85 and AUPRC ≥ 0.85). *target sequences underwent clustering and representative sequence selection. RNA motifs were obtained from cisBP-RNA except PUM2, for which the RNA motif was obtained from supplementary Table S2 of Incarnato et al., 2013 (PubMed ID: 23863844).

The positive, negative training set, and PWM for each RBP could be downloaded from http://bioinfo.vanderbilt.edu/beRBP/download/TabS1.7z .

Table S2. Whole-transcriptome target scan by beRBP.

RBP	eCLIP cell line	# eCLIP targets	# beRBP targets	# inter-section targets	PV§	Sensitivity	Specificity	Precision
FMR1*	K562	7,809	23,278	4,651	1.80E-259	0.6	0.61	0.2
FXR1	K562	8,894	4,503	1,434	2.50E-171	0.16	0.94	0.32
FXR2	K562	7,049	23,942	4,371	1.60E-263	0.62	0.6	0.18
HNRNPA1	K562	5,915	1,532	511	4.60E-132	0.09	0.98	0.33
HNRNPC	HepG2	8,676	4,739	1,942	<2.2E-16	0.22	0.94	0.41
IGF2BP1	K562	19,318	27,631	13,604	<2.2E-16	0.7	0.62	0.49
IGF2BP2	K562	18,058	17,962	8,587	<2.2E-16	0.48	0.75	0.48
IGF2BP3	HepG2	17,707	14,691	7,208	<2.2E-16	0.41	0.8	0.49
KHDRBS1	K562	10,163	89	23	0.072	0	1	0.26
LIN28B	K562	15,671	25,964	10,301	<2.2E-16	0.66	0.61	0.4
NONO*	K562	9,842	29,153	6,608	1.5E-241	0.67	0.51	0.23
PCBP2	HepG2	11,251	2,918	2,319	<2.2E-16	0.21	0.99	0.8
PTBP1*	K562	7,596	25,438	4,639	5.1E-189	0.61	0.57	0.18
PUM2	K562	15,063	19,449	9,075	<2.2E-16	0.6	0.75	0.47
RBM5*	HepG2	11,392	24,290	7,321	<1E-8	0.64	0.62	0.30
QKI	K562	4,664	13,546	2,388	<2.2E-16	0.51	0.78	0.18
RBFOX2	HepG2	6,695	4,134	1,145	3.10E-181	0.17	0.94	0.28
SF3B4*	K562	4,327	24,498	2,845	1.20E-169	0.66	0.56	0.12
SFPQ*	HepG2	2,063	22,515	1,288	7.80E-95	0.62	0.61	0.06
SRSF1*	K562	10,288	22,807	6,301	<1E-8	0.61	0.64	0.28
SRSF7*	K562	5,465	22,018	3,532	<2.2E-16	0.65	0.63	0.16
SRSF9*	HepG2	8,536	22,921	5,424	<1E-8	0.64	0.63	0.24
TAF15	K562	5,350	4,988	1,295	6.00E-278	0.24	0.93	0.26
TARDBP	K562	7,242	1,173	745	<2.2E-16	0.1	0.99	0.64
TIA1	K562	24,438	26,633	1,5169	<1E-8	0.62	0.64	0.57
U2AF2	K562	8,718	875	303	2.00E-44	0.04	0.99	0.35

*these RBPs do not have established beRBP specific models and their targets were predicted by the General model.

§ one-sided Fisher's exact test P-value for overlap between beRBP targets and eCLIP targets, as calculated in the RBPmap study (PMID: 24829458) .

Note: Targets of 17 RBPs were predicted by beRBP-Specific models (beRBP-General model results could be downloaded from http://bioinfo.vanderbilt.edu/beRBP/download/beRBP-G_targets_26RBPs.7z), whereas targets of nine RBPs (asterisk) were predicted by beRBP-General model. Totally 55,935 3'-UTRs were scanned. beRBP predictions were dichotomized by the model-specific threshold optimized in the model training process. The positive predictions concurrent with eCLIP peaks were considered as true positives, otherwise regarded as false positives. The negative predictions void of eCLIP peaks were treated as true negatives, otherwise counted as false negatives.

The 55,935 3'-UTRs, eCLIP peaks, PWM for each RBP, the threshold for each specific-model and the threshold for the General model could be downloaded from http://bioinfo.vanderbilt.edu/beRBP/download/TabS2-4.7z

Table S3. Whole-transcriptome scan performance metrics for 17 RBPs by beRBP-Specific, beRBP-General, RBPmap, and DeepBind models.

RBP	AUC (on whole eCLIP data)				AUC (on AURA-excluded eCLIP data)				AUPRC (on whole eCLIP data)				AUPRC (on AURA-excluded eCLIP data)			
	beRBP-S	beRBP-G	RBPmap	DeepBind	beRBP-S	beRBP-G	RBPmap	DeepBind	beRBP-S	beRBP-G	RBPmap	DeepBind	beRBP-S	beRBP-G	RBPmap	DeepBind
FXR1	0.6	0.63	0.54	0.61	0.57	0.6	0.53	0.6	0.23	0.24	0.16	0.18	0.17	0.18	0.14	0.16
FXR2	0.66	0.68	0.61	0.62	0.63	0.65	0.6	0.61	0.23	0.26	0.17	0.15	0.18	0.19	0.15	0.13
HNRNPA1	0.67	0.69	0.67	0.69	0.66	0.68	0.66	0.69	0.20	0.21	0.17	0.17	0.17	0.18	0.16	0.16
HNRNPC	0.71	0.69	0.63	0.73	0.67	0.66	0.6	0.71	0.30	0.27	0.22	0.27	0.21	0.20	0.17	0.21
IGF2BP1	0.72	0.72	NA	NA	0.69	0.69	NA	NA	0.59	0.57	NA	NA	0.52	0.48	NA	NA
IGF2BP2	0.65	0.66	0.58	0.59	0.63	0.63	0.58	0.58	0.48	0.47	0.35	0.34	0.42	0.40	0.30	0.29
IGF2BP3	0.65	0.67	0.6	0.62	0.63	0.64	0.59	0.61	0.47	0.47	0.35	0.34	0.42	0.41	0.30	0.29
KHDRBS1	0.55	0.64	0.61	0.64	0.55	0.64	0.61	0.64	0.21	0.25	0.22	0.22	0.20	0.25	0.22	0.22
LIN28B	0.69	0.68	0.59	NA	0.66	0.64	0.58	NA	0.48	0.47	0.32	NA	0.41	0.39	0.28	NA
PCBP2	0.76	0.77	0.76	0.65	0.7	0.71	0.75	0.64	0.51	0.53	0.40	0.26	0.31	0.32	0.33	0.21
PUM2	0.74	0.75	0.64	NA	0.72	0.73	0.63	NA	0.53	0.51	0.33	NA	0.47	0.45	0.29	NA
QKI	0.71	0.72	0.64	0.66	0.7	0.71	0.63	0.65	0.21	0.21	0.13	0.12	0.18	0.18	0.11	0.11
RBFOX2	0.68	0.65	0.65	NA	0.67	0.64	0.64	NA	0.22	0.21	0.18	NA	0.20	0.18	0.17	NA
TAF15	0.65	0.68	0.6	NA	0.6	0.63	0.6	NA	0.17	0.20	0.13	NA	0.11	0.13	0.11	NA
TARDBP	0.71	0.71	0.65	0.71	0.68	0.68	0.64	0.7	0.31	0.30	0.19	0.23	0.22	0.21	0.17	0.19
TIA1	0.681	0.690	0.683	0.680	0.655	0.667	0.672	0.676	0.62	0.62	0.54	0.52	0.56	0.56	0.40	0.48
U2AF2	0.62	0.64	0.57	0.66	0.62	0.63	0.57	0.66	0.23	0.23	0.19	0.22	0.21	0.22	0.18	0.21

Note: All 55,935 3'-UTR sequences were scanned by each method. The positive predictions concurrent with eCLIP peaks were considered as true positives, otherwise regarded as false positives. The negative predictions void of eCLIP peaks were treated as true negatives, otherwise counted as false negatives. Since each RBP-specific eCLIP dataset contains a portion of 3'-UTRs ascertained by the AURA dataset, which contributed in the training of the beRBP-Specific model, we also examined against AURA-excluded eCLIP datasets to ensure a most fair comparison between beRBP models and the competitor methods.

Table S4. Whole-transcriptome scan performance metrics for nine RBPs by beRBP-General, RBPmap, and DeepBind models.

RBP	AUC (on whole eCLIP data)			AUPRC (on whole eCLIP data)		
	beRBP-G	RBPmap	DeepBind	beRBP-G	RBPmap	DeepBind
FMR1	0.65	0.60	0.62	0.21	0.17	0.17
NONO	0.62	0.63	NA	0.24	0.24	NA
PTBP1	0.63	0.63	0.64	0.19	0.19	0.17
RBM5	0.68	0.64	0.68	0.33	0.27	0.27
SF3B4	0.64	0.54	NA	0.13	0.08	NA
SFPQ	0.66	0.61	0.63	0.07	0.05	0.05
SRSF1	0.68	0.63	0.66	0.32	0.26	0.25
SRSF7	0.69	0.62	0.67	0.21	0.18	0.16
SRSF9	0.68	0.65	0.71	0.26	0.23	0.24

Note: These 9 RBPs do not have established beRBP specific models, and therefore their targets were predicted only by the beRBP-General model.

Table S5. Wilcoxon signed rank test comparing AUCs and AUPRCs of four methods across 17 RBPs.

	AUC			AUPRC		
	median of beRBP	median of other method	p-value	median of beRBP	median of other method	p-value
beRBP Specific vs. RBPmap	0.656	0.607	0.019	0.211	0.176	0.0004
beRBP Specific vs. DeepBind	0.656	0.643	0.689	0.211	0.209	0.008
beRBP General vs. RBPmap	0.649	0.607	0.001	0.215	0.176	0.0001
beRBP General vs. DeepBind	0.649	0.643	0.259	0.215	0.209	0.0021

Note: Result here was based on AUCs and AUPRCs calculated for AURA-excluded eCLIP data (see Table S3A).

Table S6. RBP binding prediction on non-constrained RNA segments from doRiNA.

RBP	Reference	Median peak length (positive set)	# Positive sequences	# Negative sequences	AUC			
					beRBP Specific	beRBP General	DeepBind	RBPmap
ELAVL1	Kishore et al. 2011 (doi:10.1038/nmeth.1608)	9,831	499	811	0.966	**0.989**	0.967	0.955
FMR1*	Ascano et al., 2012 (DOI: 10.1038/nature11737)	4,922	401	711	NA*	**0.911**	0.775	0.907
FXR1	Ascano et al. 2012 (DOI: 10.1038/nature11737)	10,164	237	420	0.951	**0.953**	0.878	0.741
FXR2	Ascano et al. 2012 (DOI: 10.1038/nature11737)	10,411	296	491	**0.987**	**0.987**	0.929	0.823
FUS	Hoell et al. 2011 (DOI: 10.1038/nsmb.2163)	10,809	185	325	**0.968**	0.952	0.931	0.927
HNRNPC	Koenig et al. 2010 (DOI: 10.1038/nsmb.1838)	9,806	298	490	0.97	**0.979**	0.944	0.912
HNRNPL*	Shankarling et al., 2014 (DOI: 10.1128/MCB.00740-13)	12,035	197	349	NA*	**0.995**	0.929	0.908
IGF2BP2	Hafner et al. 2010 (DOI: 10.1016/j.cell.2010.03.009)	10,348	1,355	2,208	**0.979**	0.725	0.926	0.86
IGF2BP3	Hafner et al. 2010 (DOI: 10.1016/j.cell.2010.03.009)	11,125	374	602	0.973	**0.994**	0.931	0.879
LIN28A	Hafner et al. 2013 (DOI: 10.1261/rna.036491.112)	9,086	363	594	0.98	**0.993**	0.929	0.88
QKI	Hafner et al., 2010 (DOI: 10.1016/j.cell.2010.03.009)	301	1,000	2,000	0.725	**0.726**	0.597	0.68
SRSF1*	Sanford et al., 2009 (DOI: 10.1101/gr.082503.108)	8,450	103	188	NA*	**0.997**	0.939	0.93
TARDBP	Tollervey et al., 2011 (DOI: 10.1038/nn.2778)	301	4,755	5,000	0.614	0.599	**0.648**	0.566
TIA1	Wang et al., 2010 (DOI: 10.1371/journal.pbio.1000530)	9,303	428	693	0.948	**0.981**	0.928	0.912

*No beRBP Specific models are available for FMR1, HNRNPL, and SRSF1.

The positive, negative training set, and PWM for each RBP could be downloaded from http://bioinfo.vanderbilt.edu/beRBP/download/TabS6.7z .

Figure S1. ROC curves of beRBP performance by choosing different number of best matching sites.

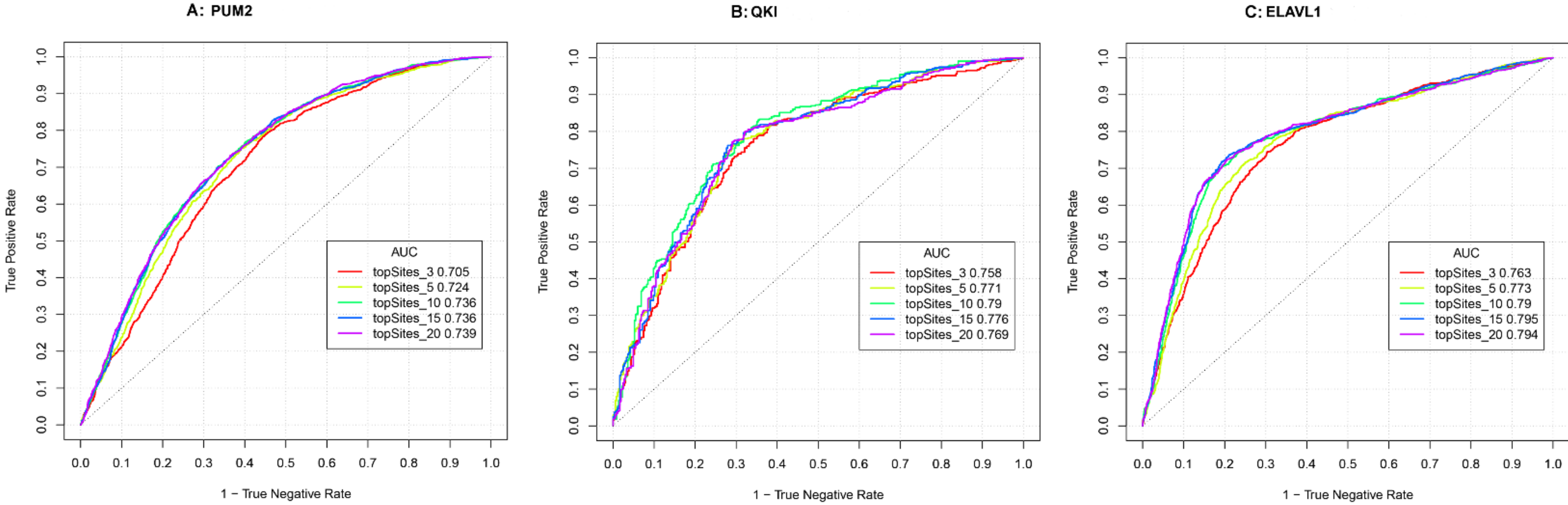

Note: The evaluation datasets, for PUM2, QKI, and ELAVL1, respectively, were compiled by authors of Oli (Livi and Blanzieri, 2014). Five different numbers of best matching sites were tested, i.e., 3, 5, 10, 15, and 20.

The positive, negative training set, and PWM for PUM2, QKI and ELAVL1 could be downloaded from http://bioinfo.vanderbilt.edu/beRBP/download/FigS1.7z .

Figure S2. Venn diagram showing the number of common RBPs with PWM available in cisBP-RNA, covered by DeepBind models, and having binding targets in AURA, eCLIP, or DoRiNA.

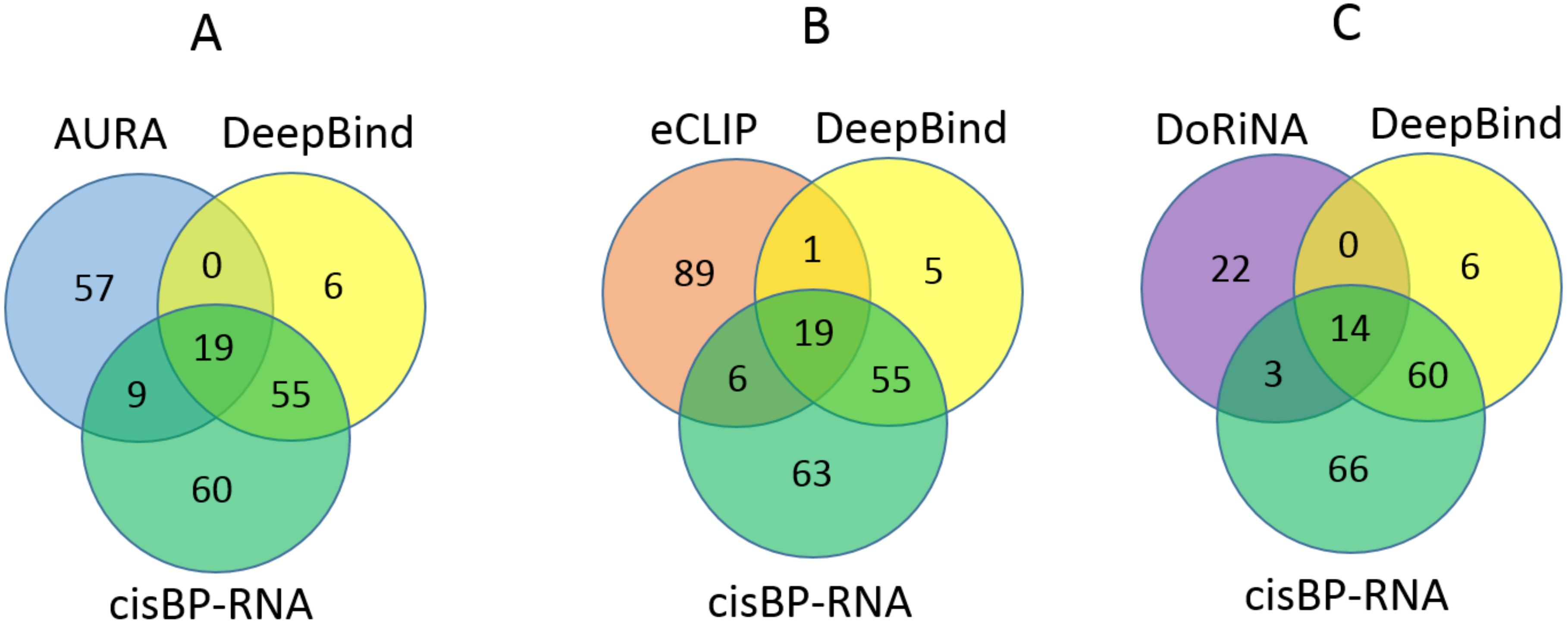

Note: whereas sub-figure A indicates a total of 28 RBPs overlap between AURA and cisBP-RNA, our Specific models encompassed 29 RBPs; whereas sub-figure B indicates a total of 25 shared RBPs between eCLIP and cisBP-RNA, our whole-transcriptome scan included 26 RBPs. The one additional RBP, PUM2, whose PWM was obtained from a published paper (PubMed ID: 23863844) but not from cisBP-RNA.

Figure S3. RBP target prediction accuracy (AUPRC) across 37 AURA benchmark datasets.

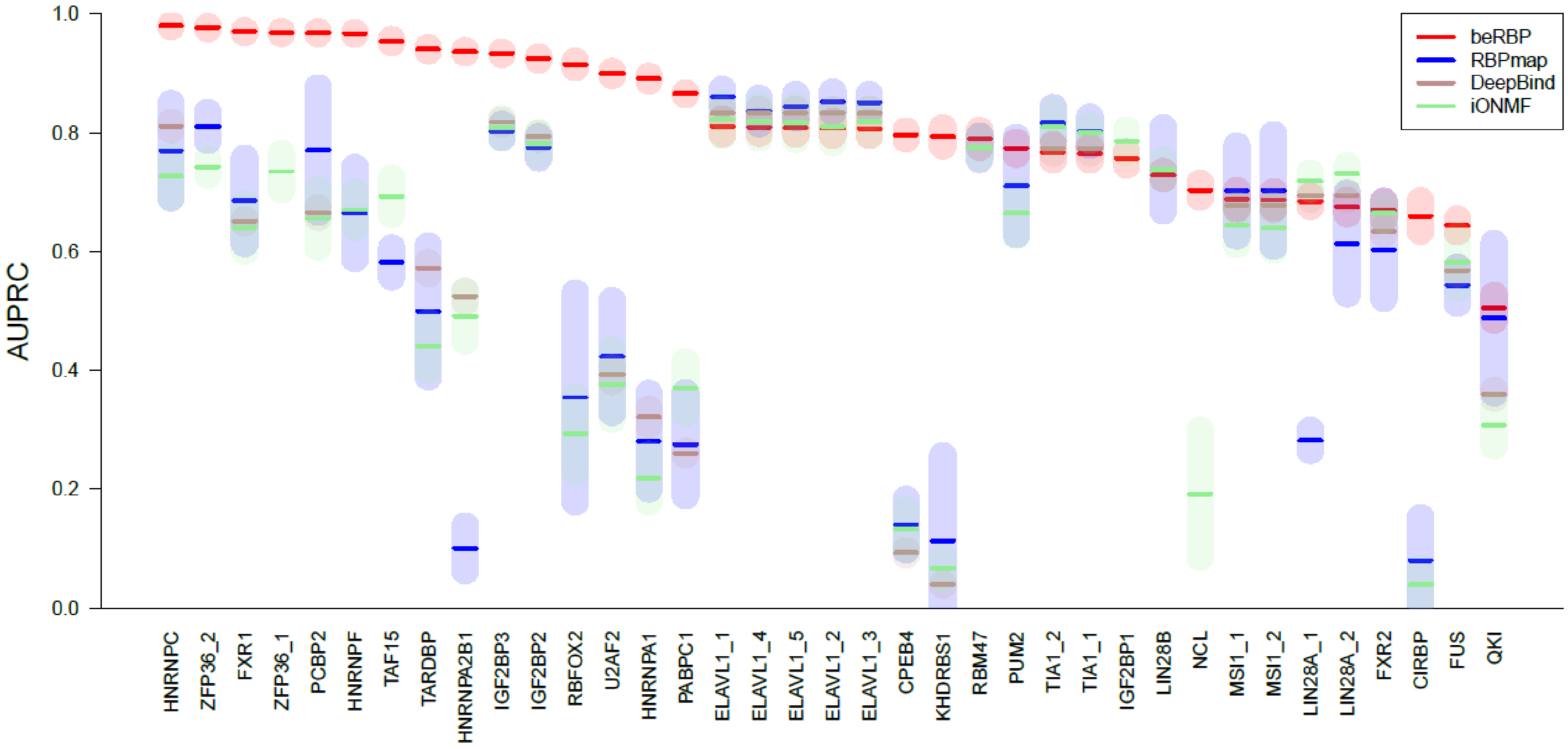

Figure S4. AUCs and AUPRCs are correlated across 37 beRBP Specific models.

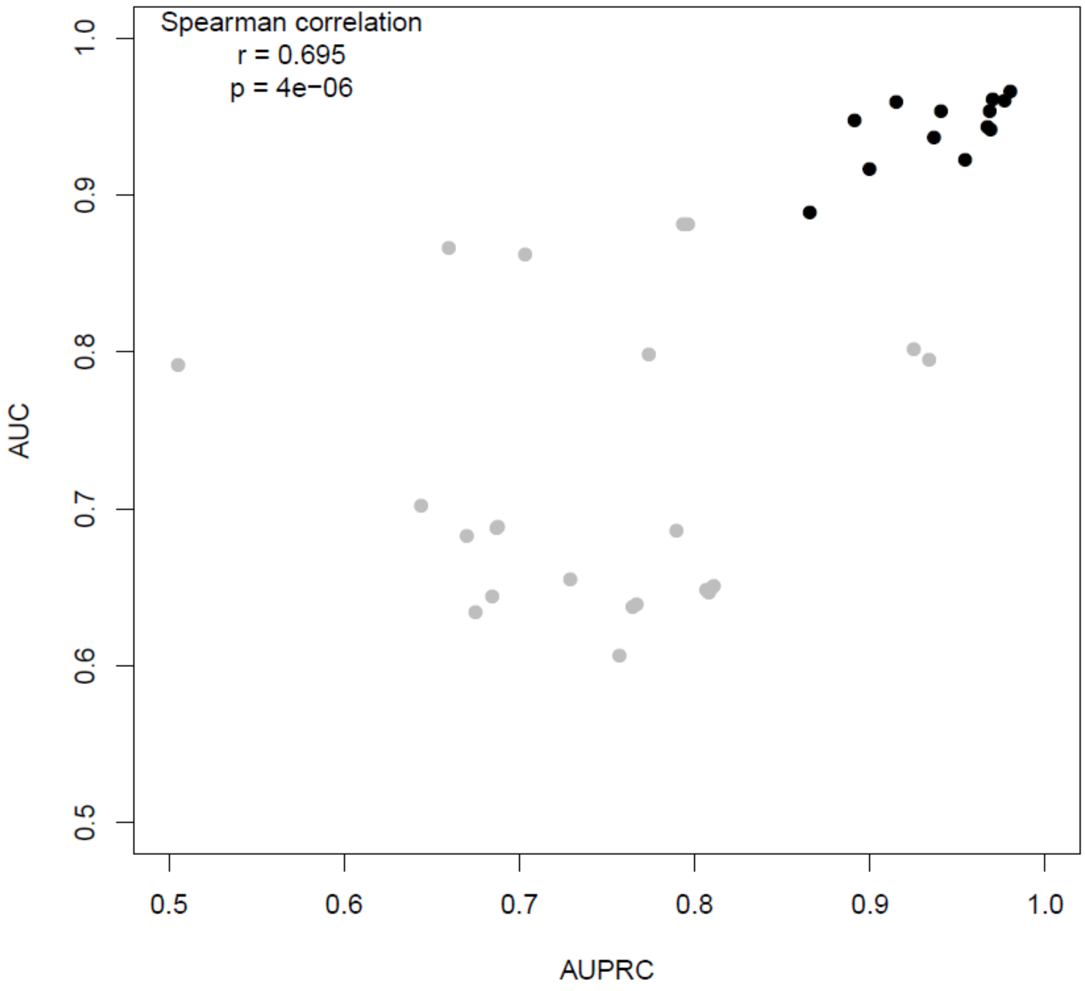

Note: dark points indicate the 13 more robust models with joint highest AUC and AUPRC.

Figure S5. Cross-prediction performance was independent of motif similarity.

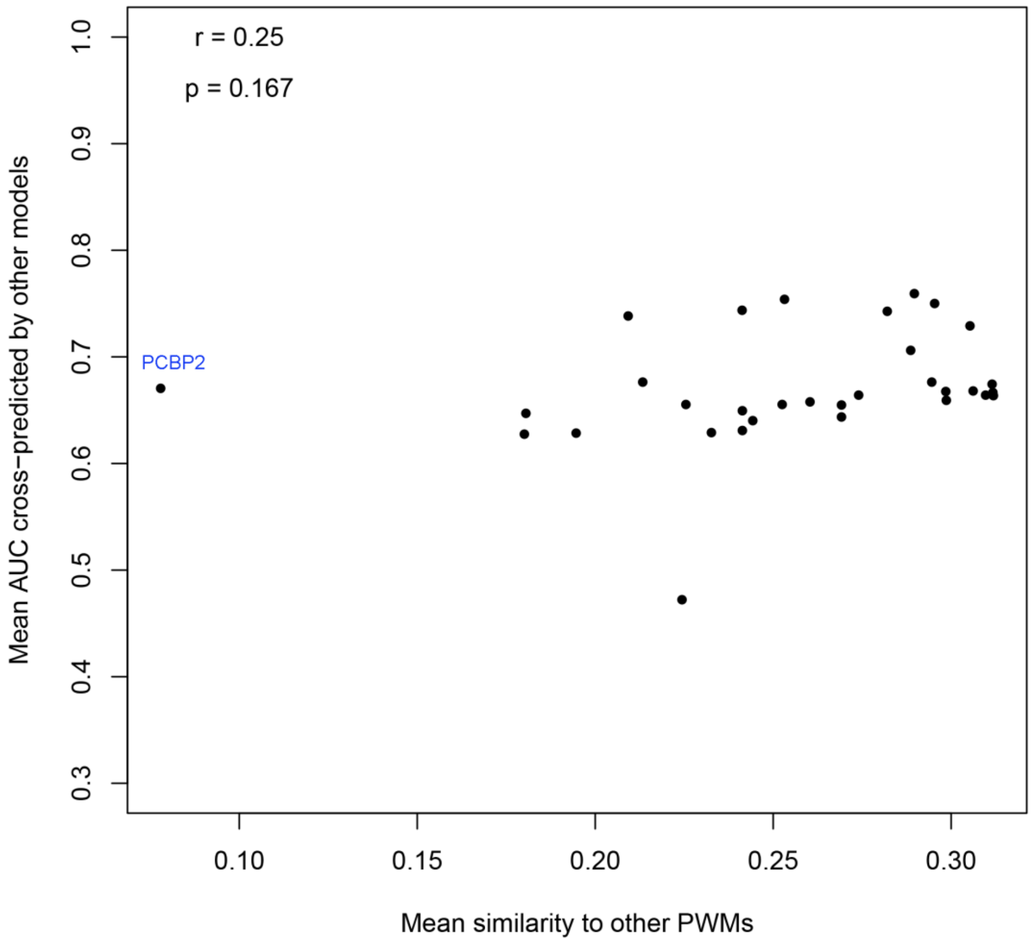

Note: X-axis is the mean similarity to other PWMs, that is, for each RBP:PWM, we calculated its average motif similarity to the other PWMs. We employed the function 'motifSimilarity' from R package PWMEnrich to calculate the similarity value between two motif matrices (PWMs). Note that we revised the function 'motifSimiliarity' not to allow reverse complement similarity calculation since we only consider the motif search on the RNA sequence. Y-axis is the mean cross-prediction performance, that is, for the model trained by each RBP:PWM, we averaged its cross-prediction performance on other RBPs. We included 33 of all 37 Specific models, excluding CIRBP, CPEB4, KHDRBS1, and NCL with few targets.

Figure S6. Negative correlation between AUC and positive dataset size of beRBP Specific models.

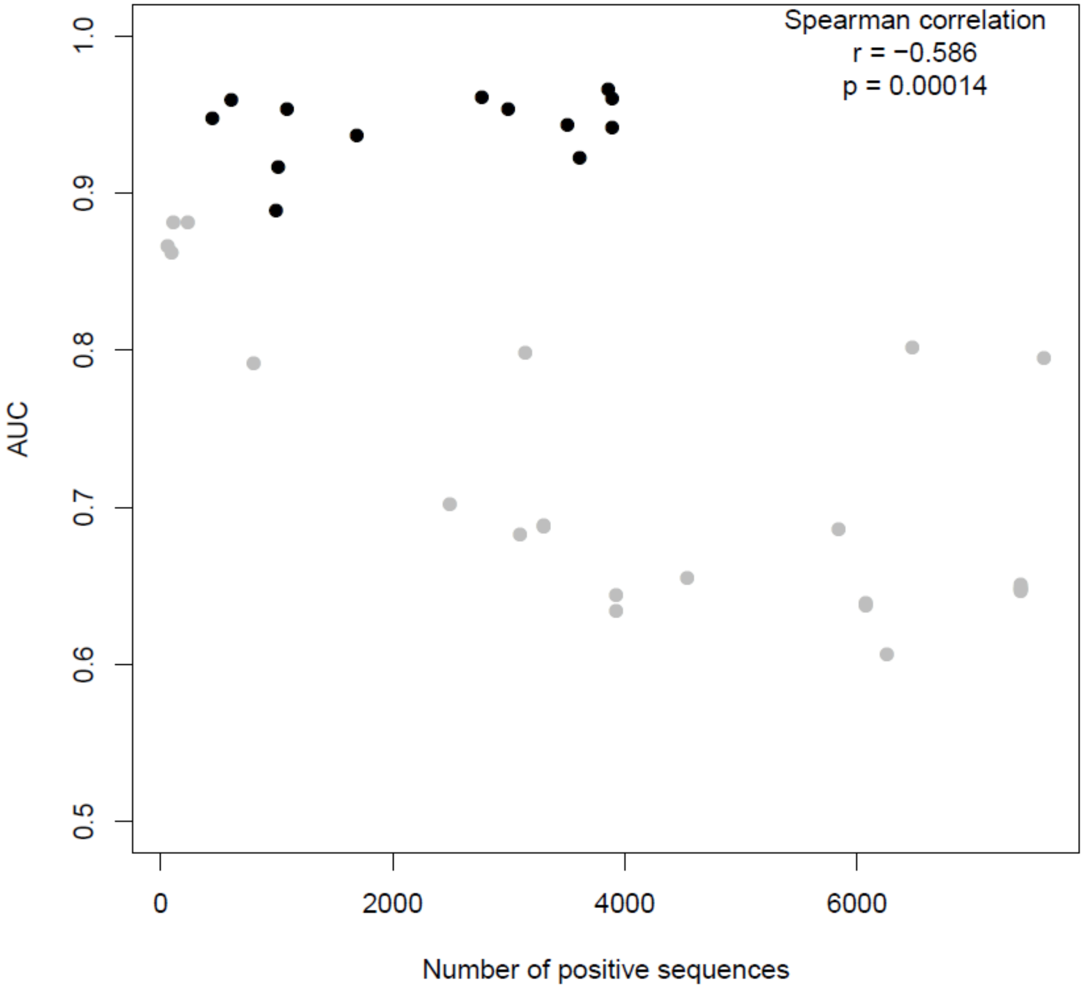

Note: dark points indicate the 13 more robust models with joint highest AUC and AUPRC.
